# Fijian sea krait behavior relates to fine‐scale environmental heterogeneity in old‐growth coastal forest: The importance of integrated land–sea management for protecting amphibious animals

**DOI:** 10.1002/ece3.8817

**Published:** 2022-04-21

**Authors:** Christopher Lowe, Gunnar Keppel, Kalisi Waqa, Stefan Peters, Robert N. Fisher, Annette Scanlon, Tamara Osborne‐Naikatini, Nunia Thomas‐Moko

**Affiliations:** ^1^ 1067 UniSA STEM University of South Australia Adelaide South Australia Australia; ^2^ 1067 Future Industries Institute University of South Australia Adelaide South Australia Australia; ^3^ NatureFiji‐MareqetiViti Suva Fiji; ^4^ U.S. Geological Survey Western Ecological Research Center San Diego California USA; ^5^ Department of Primary Industries and Regions Government of South Australia Adelaide South Australia Australia; ^6^ Faculty of Science, Technology and Environment School of Biological and Chemical Sciences The University of the South Pacific Suva Fiji; ^7^ Present address: Coastal Marine Ecosystems Research Centre Central Queensland University Gladstone Queensland Australia

**Keywords:** atoll islands, coastal forests, fine‐scale environmental heterogeneity, microclimate, microhabitats, sea krait conservation

## Abstract

The importance of terrestrial coastal ecosystems for maintaining healthy coral reef ecosystems remains understudied. Sea kraits are amphibious snakes that require healthy coral reefs for foraging, but little is known about their requirements of terrestrial habitats, where they slough their skin, digest prey, and breed. Using concurrent microclimate measurements and behavior surveys, we show that a small, topographically flat atoll in Fiji with coastal forest provides many microhabitats that relate to the behaviors of Yellow Lipped Sea Kraits, *Laticauda colubrina*. Microclimates were significantly related to canopy cover, leaf litter depth, and distance from the high‐water mark (HWM). Sea kraits were almost exclusively observed in coastal forest within 30 m of the HWM. Sloughing of skins only occurred within crevices of mature or dying trees. Resting *L. colubrina* were significantly more likely to occur at locations with higher mean diurnal temperatures, lower leaf litter depths, and shorter distances from the HWM. On Leleuvia, behavior of *L. colubrina* therefore relates to environmental heterogeneity created by old‐growth coastal forests, particularly canopy cover and crevices in mature and dead tree trunks. The importance of healthy coastal habitats, both terrestrial and marine, for *L. colubrina* suggests it could be a good flagship species for advocating integrated land‐sea management. Furthermore, our study highlights the importance of coastal forests and topographically flat atolls for biodiversity conservation. Effective conservation management of amphibious species that utilize land‐ and seascapes is therefore likely to require a holistic approach that incorporates connectivity among ecosystems and environmental heterogeneity at all relevant scales.

## Graphical abstract

We show that sea kraits are dependent on microhabitats in old‐growth coastal forest of a topographically flat Fijian atoll for terrestrial activities. Therefore, environmental heterogeneity and high‐quality terrestrial habitats, as well as healthy coral reefs, are essential for sea kraits, making them good flagship species for advocating integrated land–sea management. Therefore, a holistic approach to the conservation management of amphibious species and land‐ and seascapes is required that incorporates connectivity among ecosystems and environmental heterogeneity at all relevant scales.

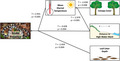

## 1 INTRODUCTION

While coral reefs have received considerable attention because they support high biodiversity and are highly vulnerable to climate change (Hughes et al., 2017; Keppel & Kavousi, 2015), associated terrestrial habitats are often an afterthought. However, there are many connections between marine and terrestrial realms that are mediated by the flow of water, transport of organic and inorganic matter, and the movement of organisms, making integrated land–sea conservation planning and management critically important (Alvarez‐Romero et al., 2011; Cicin‐Sain & Belfiore, 2005). While there has been increased interest in the concept of integrated management (Pittman & Armitage, 2016), we have limited understanding of the ecological processes linking land and sea (Alvarez‐Romero et al., 2011). Furthermore, much of the push for integrated plans has focused on improving conservation outcomes for coral reefs and marine protected areas (Cicin‐Sain & Belfiore, 2005; Klein et al., 2012), rather than looking at joint terrestrial and coral‐reef indicators.

Such integrated management should be particularly relevant for organisms that are dependent on both realms, such as sea kraits. Sea kraits (genus *Laticauda*), unlike the fully aquatic true sea snakes (subfamily *Hydrophiinae*), are amphibious (Bonnet, Ineich & Shine, 2005)—relying on marine and terrestrial coastal habitats. They diverged from closely related genera around 18 million years ago (Sanders & Lee, 2008) and have evolved adaptations to an aquatic lifestyle allowing them to spend several days foraging in marine habitats before returning to land to slough, breed, and digest prey in coastal terrestrial habitats (Shetty & Shine, 2002a). Sea kraits therefore should be a good flagship species for integrated land–sea management in the tropical Indo‐Pacific.

Sea kraits are also indicator species for ecosystem health, and the importance of healthy coral reefs for sea kraits has been repeatedly highlighted (Bonnet, Brischoux, Bonnet, Plichon & Fauvel, 2014; Brischoux & Bonnet, 2009). However, outside of New Caledonia (Bonnet & Brischoux, 2019), we know little about the terrestrial habitat requirements of sea kraits despite coastal terrestrial habitats being under increasing threat from habitat degradation and rising sea levels (Cuadros‐Casanova, Zamora, Ulrich, Seibold & Habel, 2018; Defeo et al., 2009; Nerem et al., 2018). Sea kraits are threatened by habitat loss and hunting by humans, with five of the eight species of *Laticauda* currently considered threatened (IUCN Red List Category: vulnerable; 2 species) or near threatened (Elfes et al., 2013). Declines in sea krait populations are likely having detrimental impacts on food webs and nutrients, as sea kraits are top predators on coral reefs and transfer nutrients to terrestrial environments when they defecate (Ballinger & Lake, 2006; Ineich et al., 2007).

Effective conservation of sea kraits is therefore important but will require an understanding of how they utilize terrestrial environments. In this context, fine‐scale environmental heterogeneity should be important, as animals can select locations with microclimates that provide favorable conditions for various reasons, such as thermoregulation (Huey, Peterson, Arnold & Porter, 1989; Pringle, Webb & Shine, 2003; Sears et al., 2016; Visinoni, Pernollet, Desmet, Korner‐Nievergelt & Jenni, 2015) and maintenance of water balance (Guillon, Guiller, DeNardo & Lourdais, 2013). For example, beach‐cast wrack (washed‐up algae and seagrasses) creates a mosaic of microclimates on beaches and this was found to be important for thermoregulation in shorebirds (Davis & Keppel, 2021).

We investigate the terrestrial habitat requirements of *Laticauda colubrina* on a small, topographically flat coral atoll in Fiji. We explore how the physical features (e.g., leaf litter and canopy cover) and microclimate influence *L. colubrina*, directly or through interactions. In particular, we address the following questions: (1) how much microhabitat variability is present on a coral atoll?; (2) does sea krait behavior respond to microhabitat variability?; and (3) what are the conservation implications of habitat usage by *L. colubrina*. Our results contribute to a better understanding of the terrestrial ecology of sea kraits and therefore provide the platform for improved conservation management of this species and, more generally, amphibious animals and coastal habitats.

## 2 MATERIALS AND METHODS

### 2.1 Field location and species

*Laticauda colubrina* (Yellow Lipped Sea Krait) is the most abundant and widespread of the eight sea krait species in the genus *Laticauda* (Gherghel, Papeş, Brischoux, Sahlean & Strugariu, 2016), occurring along coastlines from South‐east Asia to the Pacific, including Islands that are most at risk from rising sea levels (Becker et al., 2012). Relative to other members of *Laticauda*, *L. colubrina* is less adapted to the marine environment and as a result is comparatively better suited to the terrestrial environment (Bonnet et al., 2005).

**FIGURE 1:**
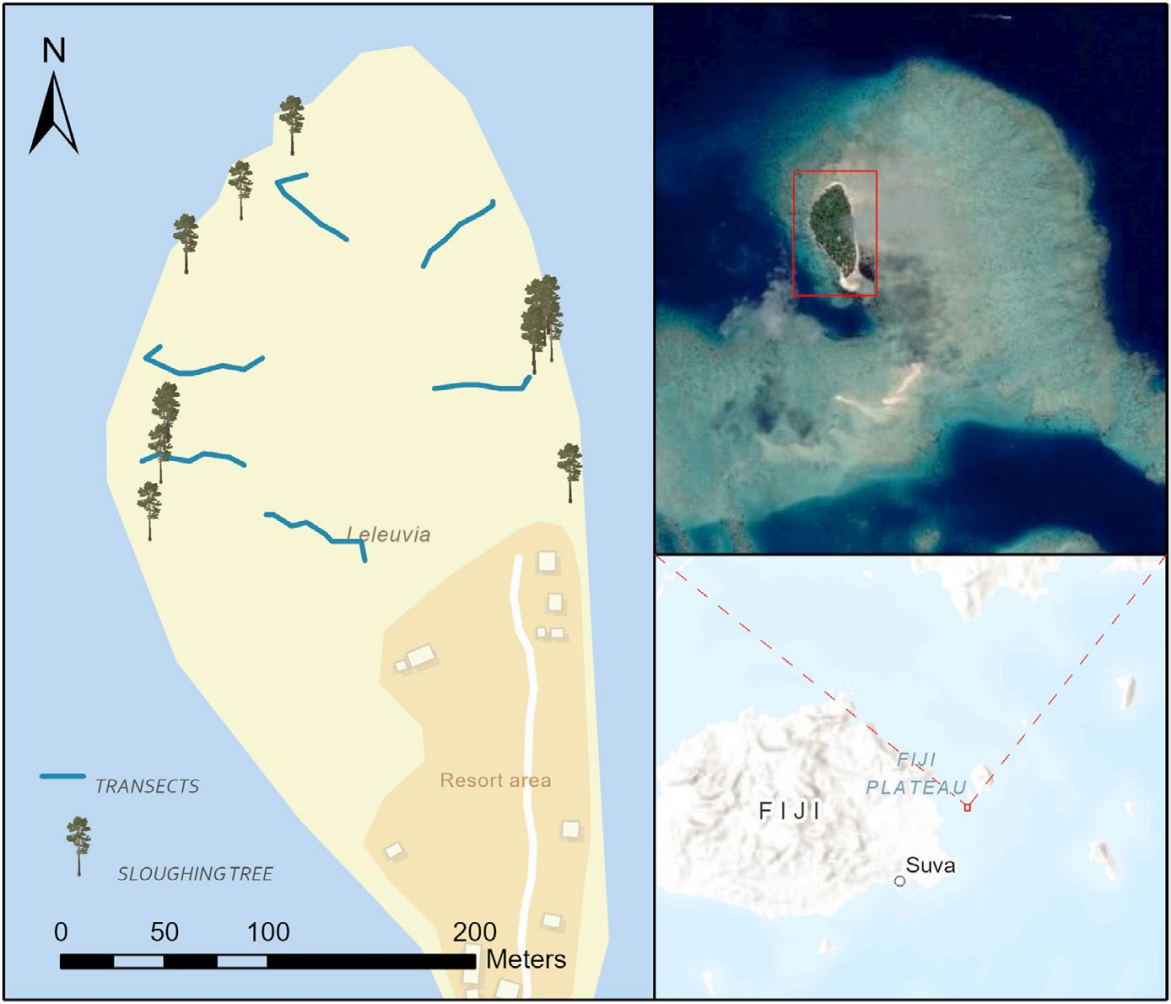
The location of Leleuvia Island in relation to Viti Levu (Fiji), the extent of extensive reef system surrounding Leleuvia Island, and the location of the eco‐resort, transects established, and trees in which sloughing individuals of *Laticauda colubrina* were found (sloughing trees) at the time of this study

**FIGURE 2:**
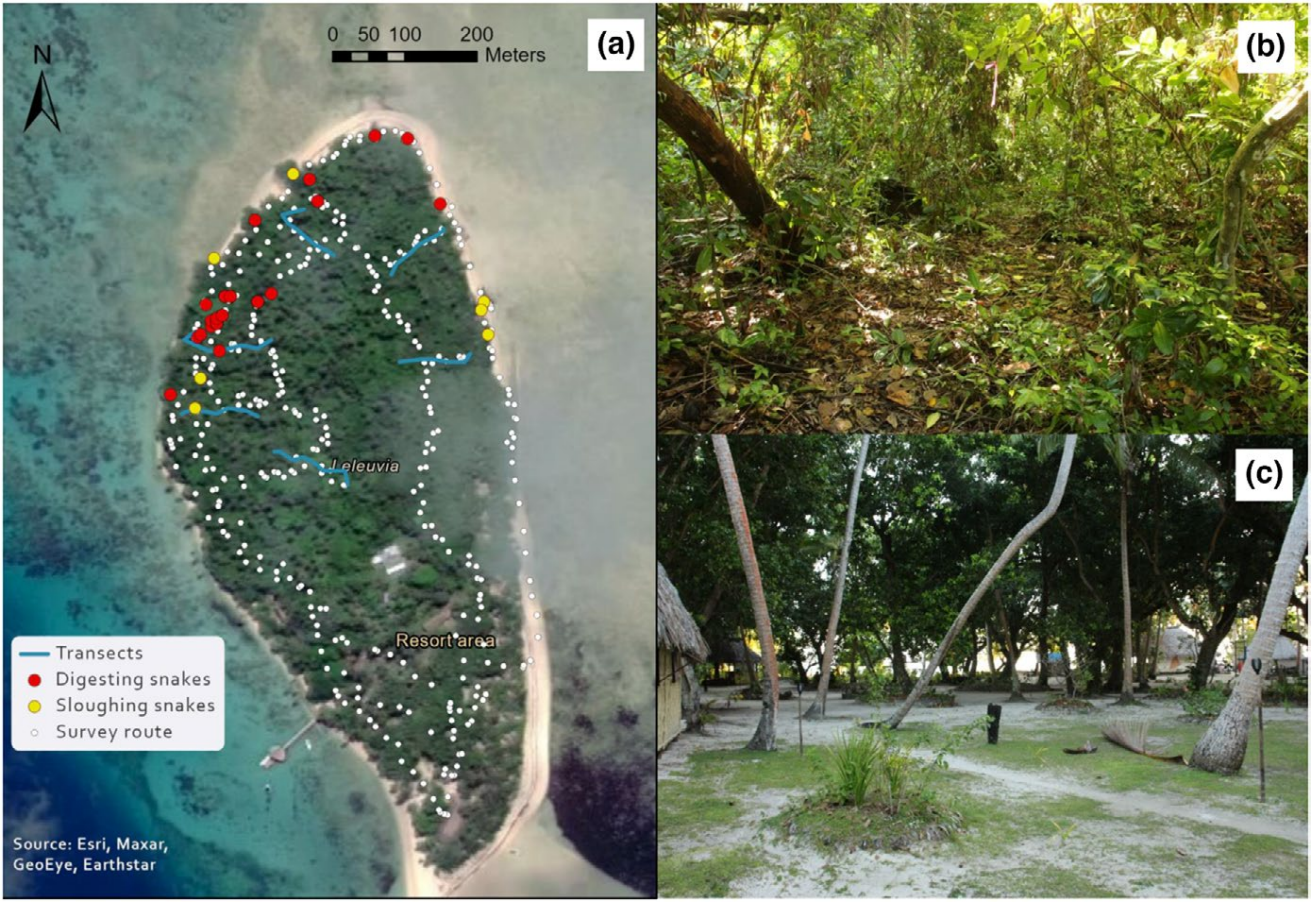
Habitats, survey sites, and sea krait locations of Leleuvia Island; (a) survey route for the 15 surveys of sea krait behavior, transects for microclimate data collection, and locations of sloughing and digesting snakes found on the survey on the 25th July 2019 starting at 21:00; (b) Understory of the coastal forest on the northern side of Leleuvia; (c) Understory around the resort area on the southern side of Leleuvia. Photographs: CL

Leleuvia Island is a small (9.7 ha), flat (maximum elevation of about 1.5 m), sandy coral atoll located 50 km northeast of Suva, Fiji (Figure 1), consisting of a relatively undisturbed coastal strand forest on the northern side of the island and a small eco‐resort on the southern side (Figure 2). Organic matter has accumulated in the central, forested part of the island, which has a very thin topsoil layer. The climate is wet tropical, with an estimated mean precipitation of at least 3050 mm year^−1^ and an average mean temperature of 25°C. The weather is influenced by the prevailing southeast trade winds, resulting in more sheltered north and west coasts. Occasional storms and cyclones can carry salt and water onto the island. In 2016, Tropical Cyclone Winston, the most severe cyclone recorded to make landfall in the Southern Hemisphere with sustained winds of 280 km h^−1^, passed just north of the island. It defoliated plants, broke branches, killed epiphytes, salinized the island's freshwater lens, eroded up to 6 m of beach on the east coast, and severely damaged resort infrastructure. However, the plant species composition changed relatively little between 2013 and 2018. (Thaman, 2018).

Leleuvia Island has a healthy population of *L. colubrina*, thanks in part to being surrounded by an extensive reef ecosystem that covers about 50 times the area of the island (Figure 1) and provides an excellent feeding ground (Lukoschek, Heatwole, Grech, Burns & Marsh, 2007; Thaman, 2018). Therefore, the island allows investigating microhabitat selection by sea kraits in the terrestrial realm and the potential effect of habitat clearing on *L. colubrina*.

### 2.2 Field methods

Six transects were established in the forested part of Leleuvia Island (Figure 2a). Five 55‐m transects were positioned perpendicular to the coast, commencing at 2‐m inland of the high‐water mark (HWM). Another 65‐m transect was established in the center of the island. Each transect was equipped with 7 (55 m transects) or 8 (65 m transect) Hygrochron DS1923 microclimate sensors (Eclo Solutions) located at 2.5, 7.5, 12.5, 22.5, 32.5, 42.5, and 52.5 m (55 m transects), and 0, 10, 20, 25, 35, 40, 50, and 60 m (65 m transect) from the starting point of the transect. These 43 sensors were supplemented with 17 additional sensors placed close to resting *L. colubrina*. Of the 60 sensors, 20 were positioned in areas with kraits present and 40 in areas without. Ten of these sensors, five each in locations with and without kraits, malfunctioned or were lost.

Microclimate sensors were standardized at room temperature for 24 h, then programmed to record temperature and humidity at 20‐min intervals for two weeks. Each sensor was positioned 30 mm (approximately body height of *L. colubrina*) above ground on a 400‐mm wooden stake of 10 mm diameter. A white paper cup, perforated with holes to facilitate airflow, was attached above the sensor and used as a radiation shield. The brand of sensor used, the material, and surface reflectivity of the radiation shield will impact microclimate measurements (Maclean et al., 2021). While self‐made radiation shields therefore have shortcomings and produce results that differ from those obtained by weather stations (Maclean et al., 2021; Terando, Youngsteadt, Meineke & Prado, 2017), they can potentially provide more realistic (as experienced by organisms) measurements (Ashcroft, 2018). Therefore, despite differing from standardized measurements, microclimate data collected using consistent shielding is comparable, and potentially more relevant (Davis & Keppel, 2021; Keppel, Anderson, Williams, Kleindorfer & O’Connell, 2017).

**FIGURE 3:**
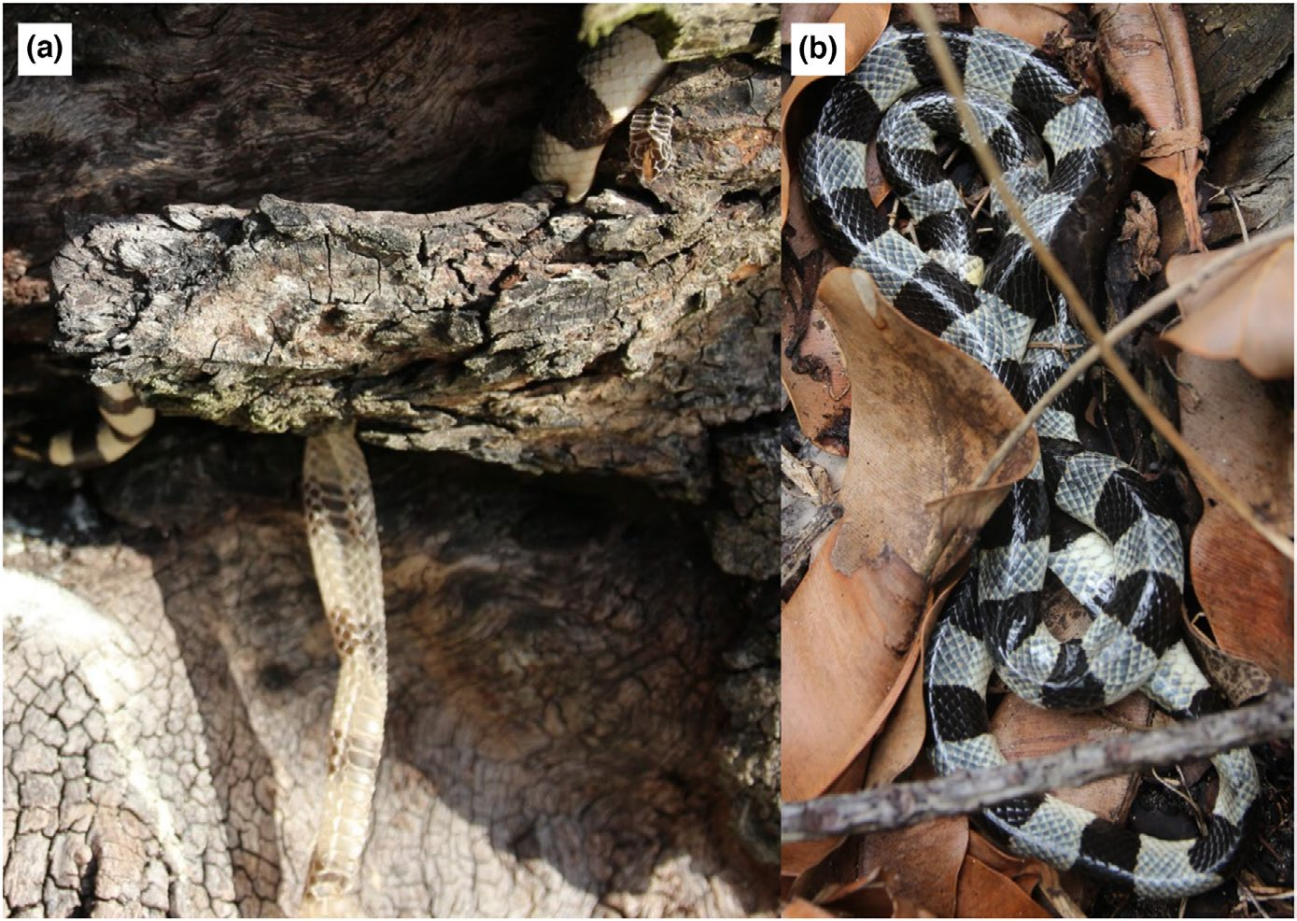
Examples of sloughing and resting habitats for *Laticauda colubrina*; (a) A sloughing individual (with shed skin) in the bark of a mature *Calophyllum inophyllum*; (b) An individual resting in the leaf litter. Photographs: CL

A survey route (Figure 2a) was established around the island to locate sea kraits. A total of 15 surveys were completed over a 9‐day period, with surveys occurring every second day (on 21, 23, 25, 27, and 29 July 2019) to minimize disturbance to the animals. Three surveys were completed on each survey day. Surveys started at 9:00, 15:00, and 21:00 h and allowed for a larger range of behaviors to be observed, given the predominantly nocturnal activity of *L. colubrina* on land (Brischoux & Bonnet, 2009). The direction of travel for each survey was alternated to minimize pseudo‐replication. Data collected on surveys included the location of each snake using a Garmin etrex 20× GPS (Garmin), the behavior exhibited by the snake (either resting, sloughing, breeding, and traveling) (Figure 3), the percentage canopy cover in a 2‐m radius above the snake, the depth of the surrounding leaf litter using a ruler, and the plant species present in a 1‐m radius around the snake. Plants were identified using literature (Thaman, 2018; Whistler, 1992).

### 2.3 Data analysis

All analyses were undertaken in the R statistical environment (R‐Core‐Team, 2020). We converted relative humidity to absolute humidity (g m^−3^), which has greater ecological relevance (Kurta, 2014), using the formula by Mander (2012): AH = (6.112 × e^[(17.67 × T) / (T + 243.5)]^ × RH × 2.1674) / (273.15 + T), where T is temperature in °C, RH is relative humidity in %, and e is the base of natural logarithms [raised to the power of the contents of the square brackets].

#### 2.3.1 Heterogeneity in the physical environment

Plant species composition was analyzed using Kruskal's nonmetric multidimensional scaling (NMDS) with the Bray–Curtis dissimilarity coefficient and related to longitude, latitude, and distance to the HWM using vector fitting, which allows quantification of the strength of relationships through the correlation coefficient (*r* ^2^) (Dargie, 1984; Kantvilas & Minchin, 1990). These analyses were implemented in the vegan package (Oksanen et al., 2020).

#### 2.3.2 Environmental characteristics and microclimates

Following O'Connell and Keppel (2016), the microclimate data (temperature and humidity) were split into diurnal and nocturnal observations and data within 1 h before and after sunrise and sunset were excluded. The data were then used to calculate the mean and variance in temperature and humidity for both diurnal and nocturnal observations. Shapiro–Wilk tests revealed non‐normal distribution for the diurnal and nocturnal data and, consequently, nonparametric tests were used in the analyses. Linear mixed effect (LME) models implemented with the *nlme* package (Pinheiro, Bates, DebRoy & Sarkar, 2022) were used to investigate relationships between microclimate and environmental variables (canopy cover, leaf litter depth, and distance to HWM), with the day of observations as a random effect.

#### 2.3.3 Microhabitat preferences

A t‐test was used to determine if the number of snakes differed significantly between the undisturbed and disturbed site of the island. To prevent any survey bias, a standardized measure of snake presence was calculated for each side by using the formula: *SP* = *N*/*D*/*A*, where *N* is the number of snakes found on each side, *D* is the proportional distance surveyed on each side, and *A* is the percentage size of each side. *D* was adjusted to allow the distances walked to reflect the proportional sizes of each side of the island using the formula: *D* = *D_S_*/*P*/*D_D_* where *D_S_* is the average distance surveyed on each side, *D_D_* is the average distance surveyed on the disturbed side, and *P* is the proportional size of each side. This dataset was then analyzed using a t‐ test to determine whether more individuals of *L. colubrina* were found on the undisturbed versus disturbed side.

LME models using the *lme4* package (Bates et al., 2019) were used to investigate the relationship between snake presence (i.e., the presence or absence of resting/digesting kraits in a location [within 2m of a microclimate sensor] and coded 1 and 0, respectively) and the microclimate variables (mean diurnal temperature, variance diurnal temperature, mean nocturnal temperature, variance nocturnal temperature, mean diurnal humidity, variance diurnal humidity, mean nocturnal humidity, and variance nocturnal humidity).

To investigate and illustrate the relationship among environmental, microclimatic, and snake presence variables, we used structural equation modeling using the package *piecewiseSEM* to implement a confirmatory path analysis of all linear models between five key variables; snake presence, mean day temperature, distance to the HWM, leaf litter depth, and canopy cover (Lefcheck, 2016). We selected the snake presence and mean day temperature of the final survey day (29th July) for this analysis, as this day had the highest number of resting krait observations. Mean diurnal temperature was selected over other correlated temperature variables because the greatest temperature differences occurred during the day.

## 3 RESULTS

### 3.1 Heterogeneity in vegetation

Plant species composition at krait locations was significantly (*r* ^2^ = .79; *p* = .001) related to longitude (Figure S1), indicating that plants on the eastern side (exposed to the south‐east trade winds) differed from those on the sheltered, western side of the island. Canonical correspondence analysis indicated that the abundance of the trees *Barringtonia asiatica*, *Mammea odorata*, and *Ochrosia oppositifolia*, and the vine *Smythea lanceolata*, were associated with the sheltered site of the island, while the trees *Pandanus tectorius* and *Phaleria disperma*, the shrub *Volkameria inermis*, and the sprawler *Ipomoea macrantha* were associated with the more exposed side (Figure S2).

Canopy cover at krait locations ranged from 5% to 80% and was not correlated with the distance to the HWM. Leaf Litter depth ranged from 2–120 mm and the interaction between canopy cover and the abundance of *Terminalia catappa*, the only deciduous tree recorded in the study, was a significant predictor (*t* = −2.24, *p* = .032) of leaf litter depth (Figure S3).

### 3.2 Effects of environmental factors on microclimates

**FIGURE 4:**
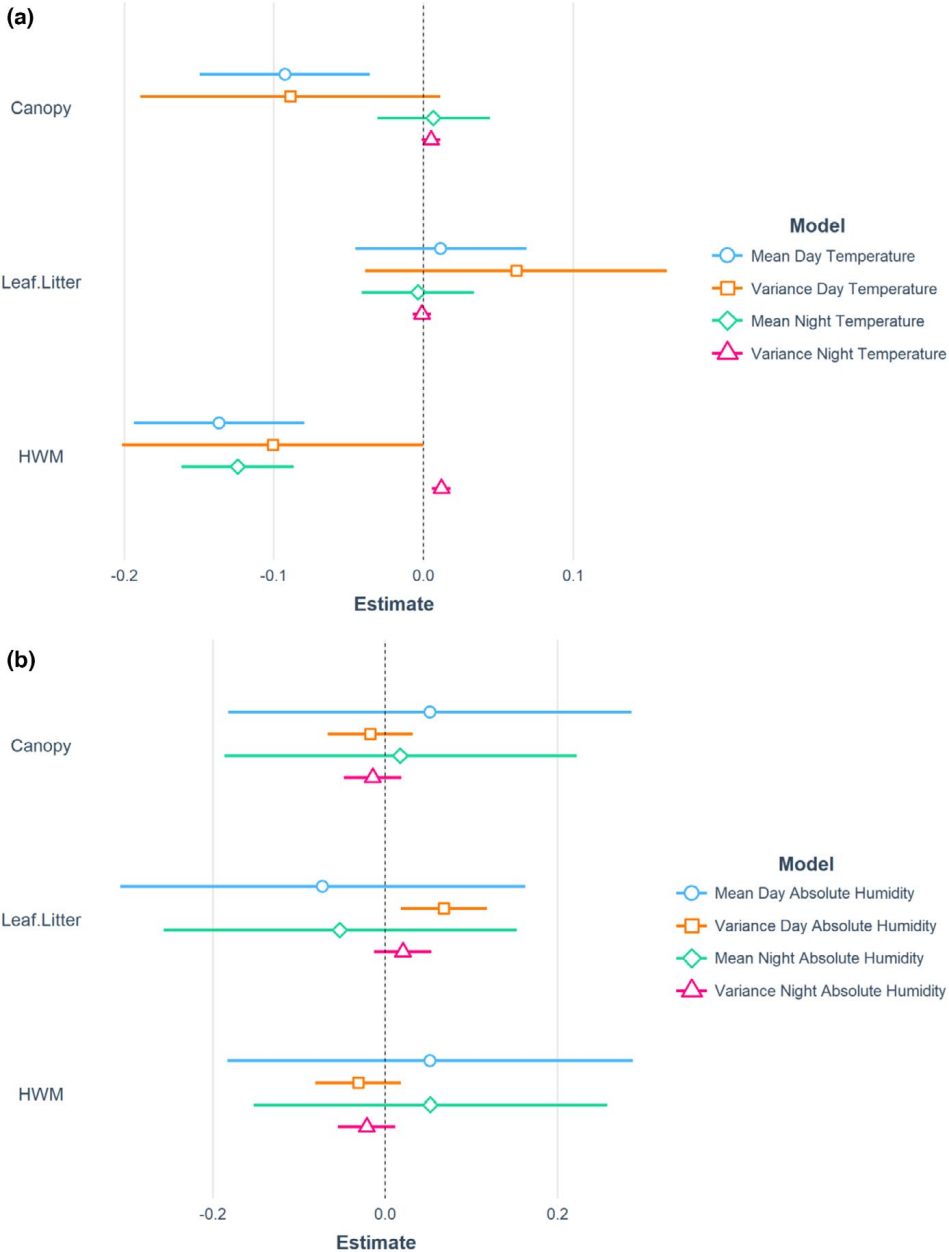
Effects of physical habitat characteristics (canopy cover, leaf litter, and distance to high‐water mark [HWM]) on (a) temperature and (b) humidity related variables. Coefficients were estimated using linear mixed‐effect models with the day of survey as a random effect. Note that there are significant interactions that are not shown for simplified illustration (see text for details). Coefficient estimates (shapes) and 95% confidence intervals (bars) are shown. Explanatory variables were scaled by mean‐centering

During the day, mean temperature ranged from 22.4–27.5°C and mean humidity ranged from 10.51–21.66 g m^−3^. Distance to the HWM and canopy cover were negatively correlated with the mean temperature (*t* = −4.84, *p* < .05 and t = −3.30, *p* = .0012, respectively; Figure 4) and the variation in temperature (*t* = −2.18, *p* = .0306 and *t* = −2.27, *p* = .0241 (as part of an interaction with leaf litter depth), respectively; Figure 4). Canopy cover only had a strong negative correlation with the variation in temperature in deep leaf litter (Figure S4a).

None of our predictors were significantly related to the mean humidity. However, the variation in humidity was significantly related to the interactions between leaf litter depth and distance to HWM and between leaf litter depth and canopy cover (*t* = −2.22, *p* = .0279 and *t* = −3.62, *p* = .0004, respectively). Canopy cover only had a strong negative correlation with the variation in humidity in deep leaf litter (Figure S4c). Leaf litter depth displayed a positive relationship with the variation in humidity near the HWM but a negative relationship furthest from the HWM (Figure S4e).

At night, mean temperatures ranged from 22.1 to 24.6°C and humidity ranged from 10.47 to 21.67 g m^−3^. None of our three predictors was significant for the variation in temperature or the variation in humidity. A three‐way interaction between canopy cover, leaf litter depth, and distance to the HWM was a significant predictor of mean temperature (*t* = −2.96, *p* = .0034). Canopy cover had a negative relationship with mean temperature in deep leaf litter and a positive relationship in shallow leaf litter, but only at greater distances from the HWM (from c. 50 m; Figure S4b).

The interactions between canopy cover and leaf litter depth and between canopy cover and distance to HWM were significant predictors of the mean humidity (*t* = −2.02, *p* = .0441 and *t* = 2.03, *p* = .0431, respectively) (Figure S4d,f, respectively). Canopy cover had a positive relationship with mean humidity in deep litter and a negative relationship at greater distances from the HWM.

#### 3.2.1 Microhabitat preferences

From 15 surveys, a total of 340 observations of *L. colubrina* were made. Of these sightings, individuals were rarely found on the disturbed site of the island, as they were significantly more likely to occur within the undisturbed areas (*t* = −6.84, *p* = 5.906 × 10^−6^). Only 2 individuals were encountered on the disturbed side, and both of these were traveling rather than resting.

**FIGURE 5:**
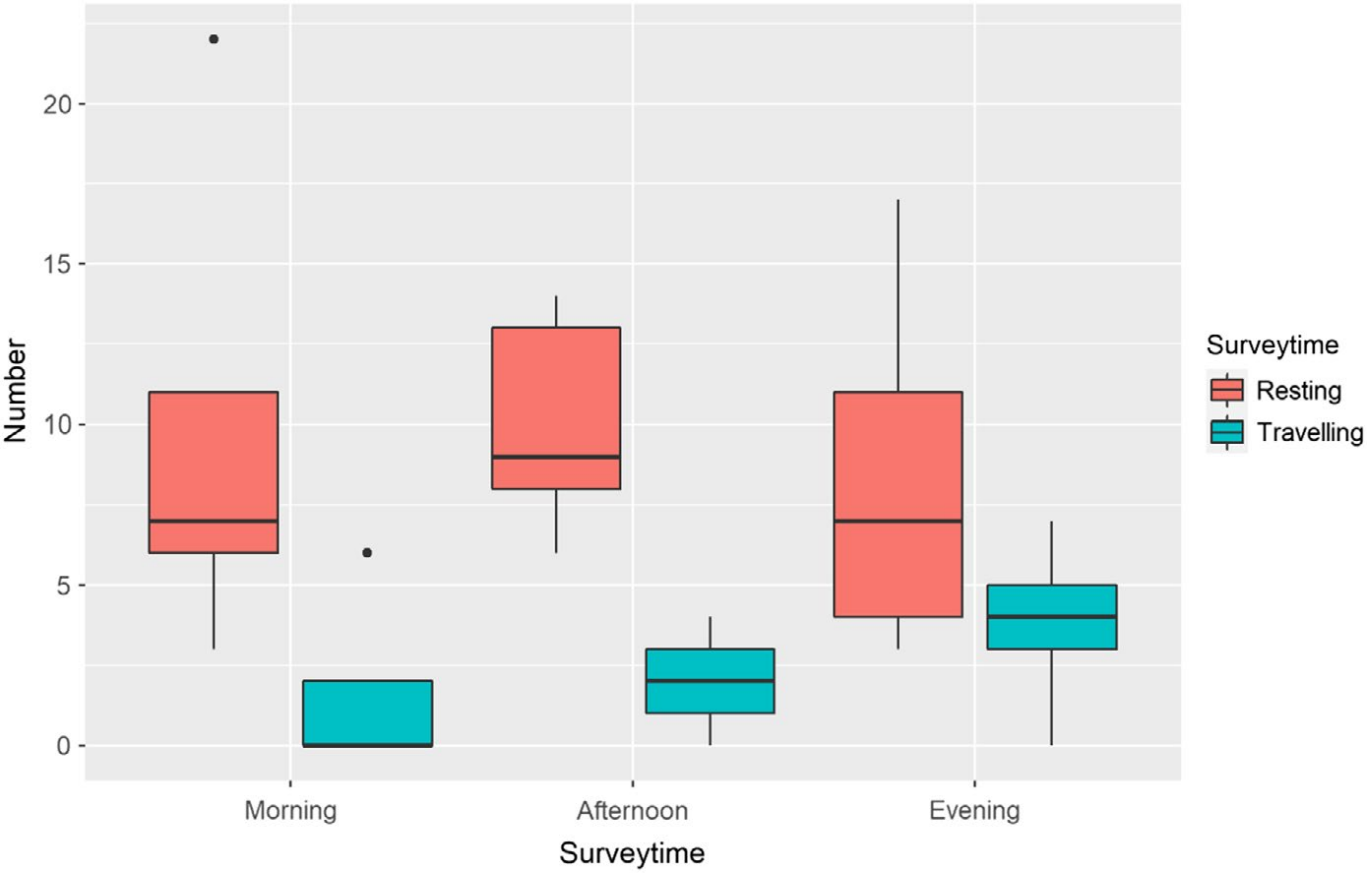
Boxplot comparing the number of resting/digesting snakes to the number of traveling individuals for each of the three survey time periods. The error bars and dots represent the minimum and maximum number of snakes found, and the boxes represent the 1st, 2nd, and 3rd quartile number of snakes found

The proportion of behaviors observed was different for the morning, afternoon, and evening survey periods (Figure 5). Traveling individuals were more frequently encountered during nocturnal surveys than in diurnal surveys. An average of 9 resting individuals, 11 sloughing individuals, and 2 traveling individuals were found in each survey. Sloughing behavior was only observed in nooks and crevices of trees along the beaches (Figure 2). Individuals that were sloughing were observed in 12 trees, which were either living trees of the thick‐barked (at old age) *Calophyllum inophyllum* and *Barringtonia asiatica*, or dead *Cocos nucifera*.

Temperature was related to the presence or absence of resting snakes, with both diurnal and nocturnal mean temperatures displaying significant, positive relationships (*t* = 5.330, *p* < .05 and *t* = 3.329, *p* = .001, respectively). The lowest temperature at which resting *L. colubrina* were found was 22.7°C during the day and 22.5°C at night. The variation in diurnal temperature was also significantly related to snake presence (*t* = 2.858, *p* = .0047), while humidity variables were not.

**FIGURE 6:**
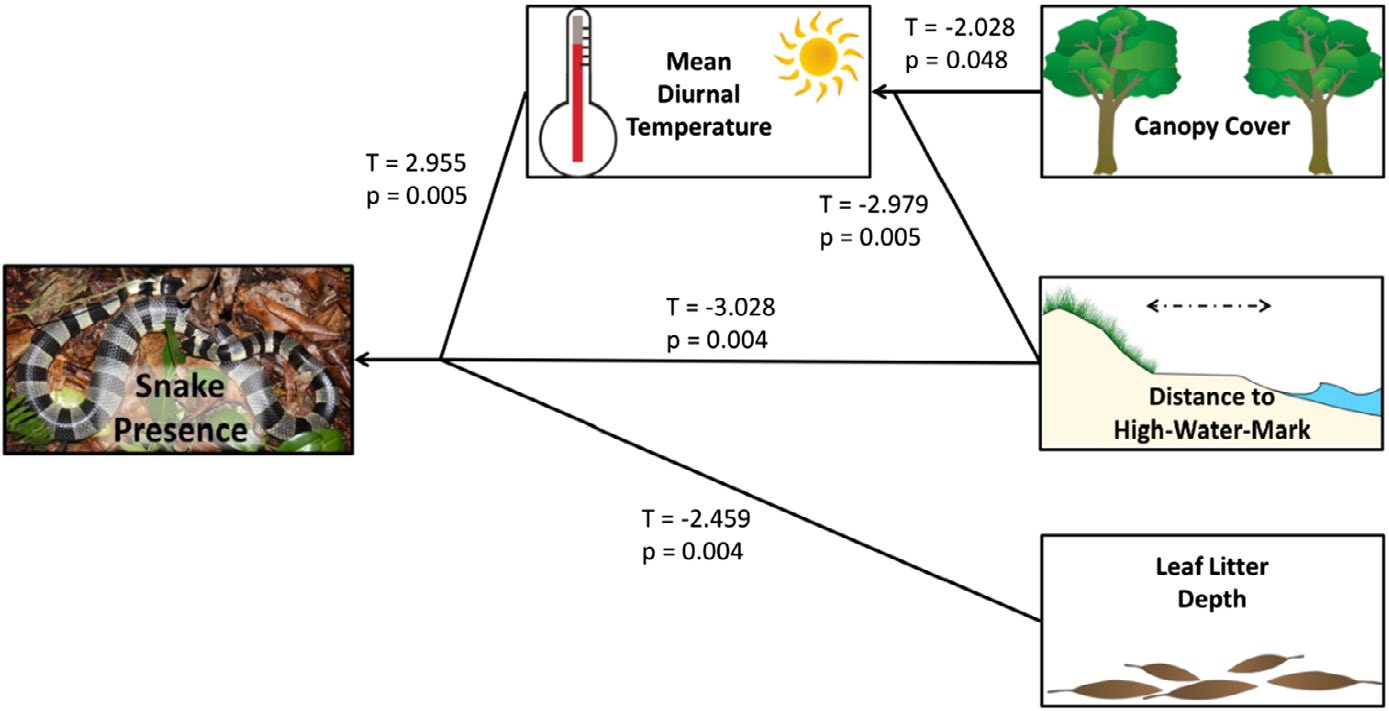
Summary of the outputs from the structural equation model. Snake occurrence (resting krait present or absent) is directly related to mean diurnal temperature, distance to high‐water mark, and leaf litter depth. Snake presence is indirectly linked to canopy cover and distance to high‐water mark, which have a significant, direct relationship with mean diurnal temperature. Image for distance to high‐water mark is modified from http://papers.risingsea.net/rolling‐easements‐2‐2‐1.html. http://papers.risingsea.net/images‐for‐rolling‐easements/figure2.jpg

Structural equation models identified the relationships among physical characteristics, microclimate, and the microhabitats selected by sea kraits to rest and digest (Figure 6). Mean diurnal temperature, leaf litter depth, and distance to the HWM were significantly related to snake presence. In addition, canopy cover and distance to the HWM were indirectly related to snake presence through their negative relationship with mean diurnal temperature.

## 4 DISCUSSION

We found that *L. colubrina* utilized specific habitats for sloughing and resting behavior. These habitats were restricted to old‐growth forest, with sloughing occurring in crevices of mature or dead trees and resting under the forest canopy at specific temperatures. Therefore, old‐growth coastal forest created considerable environmental heterogeneity that appears to be important for *L. colubrina* maintaining healthy populations, despite Leleuvia being a small island with flat topography. This implies that *L. colubrina* not only require healthy coral reef systems for hunting but may also be dependent on specific environmental conditions in their terrestrial habitats. Such conditions are found in healthy coastal vegetation, highlighting the importance of such habitats for the conservation of *L. colubrina* and other sea krait species. Because they likely require healthy marine and terrestrial habitats, sea kraits are potentially good flagship species for integrated land‐sea management.

### 4.1 Impact of terrestrial environmental conditions on snake behaviors

The environmental conditions strongly influenced the behavior of *L. colubrina*. The species generally avoided non‐forested areas and sloughing only occurred on mature and dead trees. However, the near‐absence of sea kraits from non‐forested areas could be due to either anthropogenic disturbance or the lack of essential microhabitats (Figure 2), as the presence of humans can cause shifts in animal behavior and habitat use (Bonnot et al., 2013; Gaynor, Hojnowski, Carter & Brashares, 2018). Furthermore, the location where resting behavior was displayed was, directly or indirectly, related to canopy cover and leaf litter depth. *Laticauda colubrina*, therefore, may depend on the presence of old‐growth coastal forest on sandy coral atolls. In New Caledonia, mobile beach rocks (found detached from the original substrate in the intertidal zone) were found to be a key habitat for a different sea krait species, *L. laticaudata* (Bonnet, Brischoux, Pearson & Rivalan, 2009).

Sea kraits slough at more frequent intervals compared to terrestrial snakes (Shankar & Whitaker, 2009; Shetty & Shine, 2002a) to remove attached marine organisms that would induce drag, meaning that finding suitable habitats for this behavior is likely to be important. Sea kraits have been shown to consequently use trees and other objects, such as rocks, as sloughing areas (Brischoux & Bonnet, 2009; Tyabji, Mohanty, Young & Khan, 2018). In our study, all of the sloughing locations were in the crevices of trees created by thick bark or decaying parts of the trunk. As these microhabitats are only found in mature or dying trees, a well‐developed coastal forest is likely to be an important habitat characteristic for sea kraits on sandy atolls, like Leleuvia, which do not have any large rocks.

Temperature plays a key role for microhabitat selection in animals (Davis & Keppel, 2021; Visinoni et al., 2015). It appeared to significantly influence the selection of resting sites for *L. colubrina*, with the species being found in sites with higher mean and variation in temperature, which in turn was moderated by canopy cover and distance to the HWM. Like other snake species, *L. colubrina* is ectothermic and as such environmental temperature plays an important role in allowing the individuals to thermoregulate (Huey et al., 1989; Pringle et al., 2003; Shetty & Shine, 2002a; Webb & Shine, 1998). Selecting locations with temperatures closest to their optimal operating temperature will aid in digestion, a process closely tied to body temperature in snakes (Dabruzzi, Sutton & Bennett, 2012; Greenwald & Kanter, 1979; Naulleau, 1983). Therefore, the finding that sea kraits are associated with warmer mean temperatures may be a seasonal phenomenon, as data were collected during the cooler months. During the summer months, ambient temperatures would be warmer, potentially resulting in the selection of different locations (Shetty & Shine, 2002a). Furthermore, Fijian populations of *L. colubrina* breed during summer (Heatwole, Grech, Monahan, King & Marsh, 2012), and temperature plays an important part in reptile development (Siviter et al., 2017; While et al., 2018).

The negative relationship of snake presence with leaf litter depth was somewhat surprising. Because sea kraits were clearly visible to the observer, even in deep leaf litter, this is unlikely to be artifact of potentially lowered detectability. Due to their marine adaptations, sea kraits experience higher evaporative water losses compared to terrestrial snakes (Lillywhite, Menon, Menon, Sheehy & Tu, 2009) and as such would be expected to favor areas of high humidity (Guillon et al., 2013). Greater amounts of leaf litter can locally increase humidity (Barrientos, 2012; Oliveira, Pralon, Coco, Pagotto & Rocha, 2013), but no relationship between humidity and snake presence was observed, which may be due to the generally high humidity (≥10.5 g m^−3^) in our study. Upon closer inspection of the data (Figure S5), the negative relationship between leaf litter depth and snake presence seemed to be mostly driven by sea kraits being absent at leaf litter depths >60 cm. Therefore, sea kraits may be avoiding very deep leaf litter that could cover them entirely and block out solar radiation, which may assist with digestion. Comparing sea krait behavior observed here with that of the warmer summer months could shed further light on the potential role of leaf litter.

In this study, *L. colubrina* was predominately found close to the beach. This could be a result of an imperfect adaptation, although the species is better adapted to terrestrial environments than congeners (Brischoux, Tingley, Shine & Lillywhite, 2013). Sea kraits have different scale and muscular structures when compared to terrestrial snakes (Jayne, 1982, 1988). These adaptations assist in marine locomotion but hinder movement on land (Brischoux, Kato, Ropert‐Coudert & Shine, 2010; Wang, Lillywhite & Tu, 2013). More terrestrial members of the genus *Laticuada* may travel further inland compared to more aquatic species (Brischoux & Bonnet, 2009; Tyabji et al., 2018), as they are better adapted to energy efficient movement on land. Therefore, sea kraits may be avoiding travel further than necessary to reach locations with conditions suitable for digesting prey, thereby reducing their energy expenditure. There may also be behavioral differences depending on the sex or size of individuals (Shetty & Shine, 2002a; Tyabji et al., 2018) and future studies could investigate this.

*Laticauda colubrina* was more active during dusk, dawn, and at night (Figure 5), which is a likely behavioral adaptation to counter evaporative water loss, as sea kraits are more vulnerable to desiccation than terrestrial snakes due to greater skin permeability (Lillywhite et al., 2009). This disadvantage in terrestrial situations may influence strategies employed for thermoregulation and maintaining water balance. A by‐product of night and twilight activity is that the snakes are less likely to be predated on by avian predators, which are mostly diurnal hunters (Brischoux & Bonnet, 2009). Furthermore, some sea kraits have been shown to find shelter closer to the water to reduce the risk of dehydration (Heatwole et al., 2012).

### 4.2 Environmental heterogeneity on a flat coral atoll

Fine‐scale environmental heterogeneity is important for animals, but we are just beginning to understand the complexity of interactions between physical features (e.g., topography, canopy cover), microclimate (temperature humidity as experienced by organisms), and the behavior of organisms. While our understanding of how physical characteristics are influencing microclimates (De Frenne et al., 2019) is improving, we are only beginning to quantify the complex interactions that create the observed spatial and temporal heterogeneity in microclimates and how organisms respond to this variability (Davis & Keppel, 2021; Sears et al., 2016).

Vegetation introduced considerable environmental heterogeneity to the topographically flat atoll. Canopy cover was variable throughout the island, and the composition of plant communities differed between the exposed (to the South‐East trade winds) and the sheltered sides of Leleuvia. Differences in plant species composition may, at least partially, be related to wind tolerance as species with greater wind tolerance, such as *Pandanus tectorius* (Thomson, Doran & Clarke, 2018), were more prevalent on the exposed side. Species identity can influence the physical characteristics of the environment. We found that *Terminalia catappa*, which is briefly deciduous during the dry season (Thomson et al., 2018), locally increased the amount of leaf litter. Furthermore, the amount of canopy cover would be reduced during its leafless period.

Although this is, to our knowledge, the first study documenting microclimates in a littoral forest, the tendencies of temperature in relation to vegetation structure were similar to those observed for non‐coastal tropical forests. Cooler more stable temperatures were associated with greater canopy cover, with the capacity of forest canopies to buffer ambient temperature being well documented (De Frenne et al., 2019; Keppel et al., 2017). Similarly, the tendency for the mean and variation of diurnal temperature inside forests to decrease with distance from the forest edge is well established (Didham & Lawton, 1999; Ibanez, Hély & Gaucherel, 2013), and we observed such trends with distance from the HWM (Figure 4). Therefore, the edges of coastal forests seem to share similar microclimatic tendencies to those of non‐coastal terrestrial forests, although studies focused specifically on microclimate should be undertaken to confirm this.

Humidity data are less often reported than temperature but can vary considerably (Anderson et al., 2018). In our study, absolute humidity had complex relationships with the environmental parameters measure, which is similar to results from other studies (Anderson et al., 2018). The depth of leaf litter was a significant predictor of humidity when interacting with distance to the HWM and canopy cover. Greater amounts of leaf litter are known to increase humidity (Oliveira et al., 2013). Furthermore, greater canopy cover reduces the amount of photodegradation that can occur (King, Brandt & Adair, 2012; Marinho, Martinelli, Duarte‐Neto, Mazzi & King, 2020), potentially increasing the build‐up of leaf litter and that, in turn, should increase humidity (Barrientos, 2012).

### 4.3 Conservation implications

Our findings suggest that healthy, mature forests with good canopy cover, old trees with cavities, a variety of leaf litter depths, and extending at least tens of meters from the high‐water mark provide important environmental heterogeneity that may be required for sea kraits to exhibit a range of essential terrestrial behaviors, such as digesting their food and sloughing their skin. However, coastal forests are highly threatened by both global warming and human development (Nerem et al., 2018). In the tropics, they are intrinsically connected with coral reefs (Fernando, Samarawickrama, Balasubramanian, Hettiarachchi & Voropayev, 2008; McPhee‐Shaw et al., 2007).

The importance of both, healthy coastal forest and healthy coral reefs, for *L. colubrina* suggests that integrated management of these two habitats could be most effective for conservation and that sea kraits and other amphibious species may be good flagship species for integrated land–sea management in general. Sea kraits also play a key role in connecting these habitats, being a top predator of the coral reef ecosystems and transferring nutrients from the ocean to the land through defecation and the sloughing of skin (Ballinger & Lake, 2006). This suggests that the status and health of sea krait populations may provide an indicator for the quality of coastal habitats, both the terrestrial and marine components.

Fine‐scale environmental heterogeneity is essential to allow mobile organisms access to suitable habitats for different activities and under varying environmental conditions (Davis & Keppel, 2021; Visinoni et al., 2015). Despite being extremely important ecologically and for the persistence of populations, fine‐scale environmental heterogeneity is often underappreciated and hence is more likely to be overlooked in conservation planning (Alvarez‐Romero et al., 2011; Davis & Keppel, 2021; Hunter et al., 2017; Sears et al., 2016). However, a holistic understanding of habitat requirements at different scales appears to be essential for effective conservation management.

Sea krait conservation is further complicated by sea kraits being extremely philopatric (Brischoux, Bonnet & Pinaud, 2009; Shetty & Shine, 2002), not straying from their home reef and associated habitats. As a result, populations can be genetically and phenotypically highly differentiated, although the generality and complexity of these patterns requires further investigation (Bech et al., 2016; Tandavanitj, Ota, Cheng & Toda, 2013). Therefore, protecting a single population may be insufficient to maintain the variation existing in the species and a global approach to protecting remaining coastal forests associated with coral reefs is likely to be most successful.

## 5 CONCLUSIONS

Our study highlights the relationship between the terrestrial behaviors of sea kraits and environmental heterogeneity linked to old‐growth forest. Some observed behaviors appear to be influenced by snakes being ectotherms (e.g., selection of warmer locations for resting) or being amphibious (e.g., limited travel during the day due to limited adaptation to terrestrial environments). Other behaviors appeared to be dependent on the presence of suitable habitat, such as crevices to assist with the sloughing of skins. Further microhabitat requirements would likely be detected if the study was extended to other seasons and extreme weather events. Hence, different aspects of environmental heterogeneity created by old‐growth forests are likely to be important for sea kraits to effectively undertake their terrestrial behaviors during different weather conditions.

Maintaining healthy populations of sea kraits is therefore complex. In addition to healthy reefs, adjacent terrestrial habitats with enough environmental heterogeneity seem to be important. In our study system, the necessary environmental heterogeneity was only provided by healthy and mature coastal forest. Given ongoing degradation of both reefs and coastal forests, this suggests that many sea krait populations may currently be highly stressed and threatened and that integrated land‐sea management plans could best ensure their persistence.

## CONFLICT OF INTEREST

The authors have no conflicts of interest.

## AUTHOR CONTRIBUTIONS

**Christopher Lowe:** Conceptualization (equal); Data curation (equal); Formal analysis (lead); Investigation (lead); Methodology (equal); Project administration (lead); Supervision (equal); Validation (lead); Visualization (lead); Writing – original draft (equal); Writing – review & editing (equal). **Gunnar Keppel:** Conceptualization (equal); Data curation (equal); Formal analysis (supporting); Funding acquisition (lead); Investigation (supporting); Methodology (equal); Project administration (supporting); Resources (equal); Supervision (equal); Validation (supporting); Visualization (supporting); Writing – original draft (equal); Writing – review & editing (equal). **Kalisi Waqa:** Investigation (supporting); Resources (supporting); Writing – review & editing (supporting). **Stefan Peters:** Visualization (supporting); Writing – review & editing (supporting). **Robert N. Fisher:** Methodology (supporting); Writing – review & editing (equal). **Annette Scanlon:** Supervision (supporting); Writing – review & editing (supporting). **Tamara Osborne‐Naikatini:** Methodology (supporting); Writing – review & editing (equal). **Nunia Thomas‐Moko:** Funding acquisition (supporting); Project administration (supporting); Resources (equal); Supervision (supporting); Writing – review & editing (supporting).

## Supporting information

Appendix S1

## Data Availability

Data is available in Dryad (https://doi.org/10.5061/dryad.3n5tb2rkn).
