# Supplementary material for: Fijian sea krait behavior relates to fine‐scale environmental heterogeneity in old‐growth coastal forest: The importance of integrated land–sea management for protecting amphibious animals
Source: Ecol Evol. 2022 Apr 21;12(4):e8817. doi: 10.1002/ece3.8817 (PMC9022443; doi:10.1002/ece3.8817)
Supplement: Supplementary file 1 — Appendix S1 [file ECE3-12-e8817-s001.doc]

**Appendix S1: Vegetation Analyses**

**Methods**

*Multivariate Analysis*

We used the species composition data collected for all 38 vegetation points to conduct Kruskal’s non‐metric multidimensional scaling (NMDS) based on the Bray–Curtis dissimilarity coefficient in the *vegan* package. We then related the plotted sites on the NMDS plot to the longitude, latitude and distance to the high-water mark for each transect point using vector fitting, which allows quantification of the strength of relationships through the correlation coefficient (*r*^2^) (Dargie 1984, Kantvilas and Minchin 1990). Using Canonical Correspondence Analysis (ter Braak 1986) implemented in the *vegan* and *labdsv* packages, we visualised affinities of plant species that occurred at three sample points with regard to distance to the high water mark and longitude.

We tested what determined the depth of leaf litter by relating leaf litter depth to percentage canopy cover, distance from the high-water mark and the abundance (as measured on a scale of 1-5: 1 = <10% cover, 2 = 10-24.9%, 3 = 25-49.9%, 4 = 50-74.9%, 5 ≥ 75%) of *Terminalia catappa*, a common deciduous species. We simplified a linear model including all three explanatory variables and their interactions by eliminating all non-significant interactions and variables. We illustrated the significant interaction between Terminalia abundance and canopy cover using the *jtools*, *ggplot2* and *interactions* packages.

**Results**

*Heterogeneity in the Physical Environment*

Plant species composition at transect points, as represented by non-metric multidimensional scaling (NMDS), was significantly (*r*^2^ = 0.79; *p* = 0.001) related to longitude (Fig. 1), indicating that the eastern side that is exposed to the south-east tradewinds and the more sheltered, western side of the island differed in species composition. Canonical correspondence analysis indicated that the trees *Mammea odorata* and *Ochrosia oppositifolia*, the vine *Smythea lanceolata*, and the epiphytic fern *Davallia solida* were associated with the sheltered site of the island, while the trees *Pandanus tectorius* and *Phaleria disperma* the shrub *Volkameria inermis*, the vine *Parsonisa laevis* and the sprawler *Ipomoea macrantha* were associated with the more exposed side. The tree *Intsia bijuga*, the vine *Abrus precatorius* and the herbaceous *Tacca leontopetaloides*, were mostly found towards the centre of the island (Fig. 2).

*Leaf Litter Depth*

The interaction between canopy cover and the abundance of *Terminalia catappa* was a significant predictor (*t* = -2.24, *p* = 0.032) of leaf litter depth (Fig. 3).


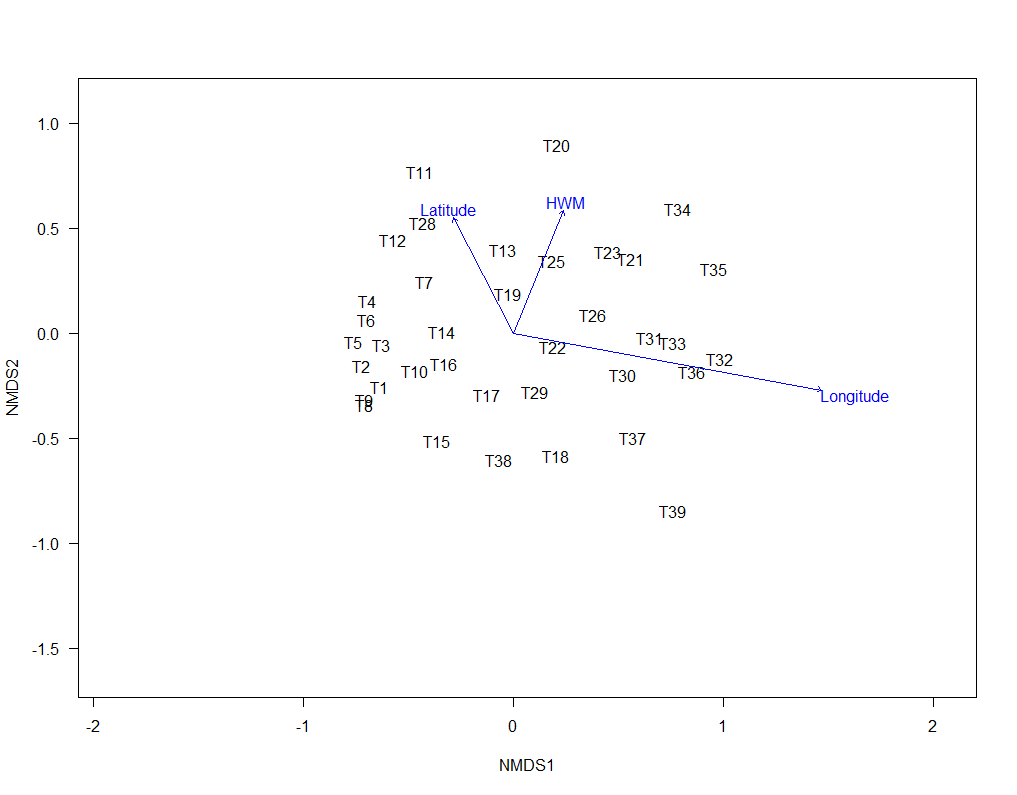


Fig. S1. Non‐metric multidimensional scaling (NMDS) for plant species at 38 transect sites on Leluvia Island, Fiji, with fitted vectors longitude (*r*^2^ = 0.79; *p* = 0.001), latitude (*r*^2^ = 0.14, *p* = 0.072) and distance from the high water mark (HWM; *r*^2^ = 0.14, *p* = 0.076). Length of vector indicates strength of correlation between vector and NMDS plot co-ordinates.


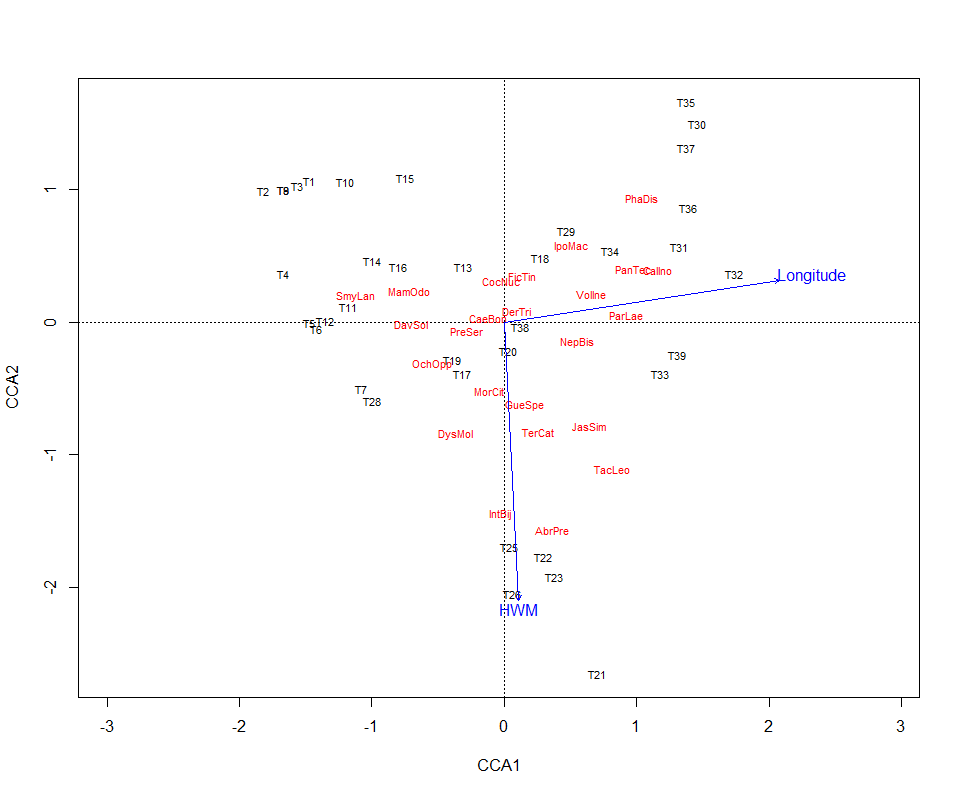


Fig. S2. Canonical Correspondence Analysis triplot showing effects of longitude (Long), latitude (lat) and distance to high-water mark (HWM) on plant community composition at 38 transect points on Leluvia Island. Species codes: AbrPre = *Abrus precatorius* L., CaeBon = *Caesalpinia bonduc* (L.) Roxb., CalIno = *Calophyllum inophyllum* L., CocNuc = *Cocos nucifera* L., DavSol = *Davallia solida* (G. Forst.) Sw., DerTri = *Derris trifoliata* Lour., DysMol = *Dysoxylum mollissimum* subsp. *molle* (Miq.) Mabb., FicTin = *Ficus tinctoria* G.Forst., GueSpe = *Guettarda speciosa* L., IntBij = *Intsia bijuga* (Colebr.) Kuntze, IpoMac = *Ipomoea macrantha* Roem. & Schult., JasSim = *Jasminum simplicifolium* G.Forst., MamOdo = *Mammea odorata* Kosterm., MorCit = *Morinda citrifolia* L., NepBis = *Nephrolepis biserrata* (Sw.) Schott, OchOpp = *Ochrosia oppositifolia* (Lam.) K.Schum., PanTec = *Pandanus tectorius* Parkinson ex Du Roi, ParLae = *Parsonsia laevis* (A.Gray) Markgr., PhaDis = *Phaleria disperma* (G. Forst.) Baill.; PreSer = *Premna serratifolia* L., SmyLan = *Smythea lanceata* Summerh., TacLeo = *Tacca leontopetaloides* (L.) Kuntze, TerCat = *Terminalia catappa* L., VolIne = *Volkameria inermis* L.


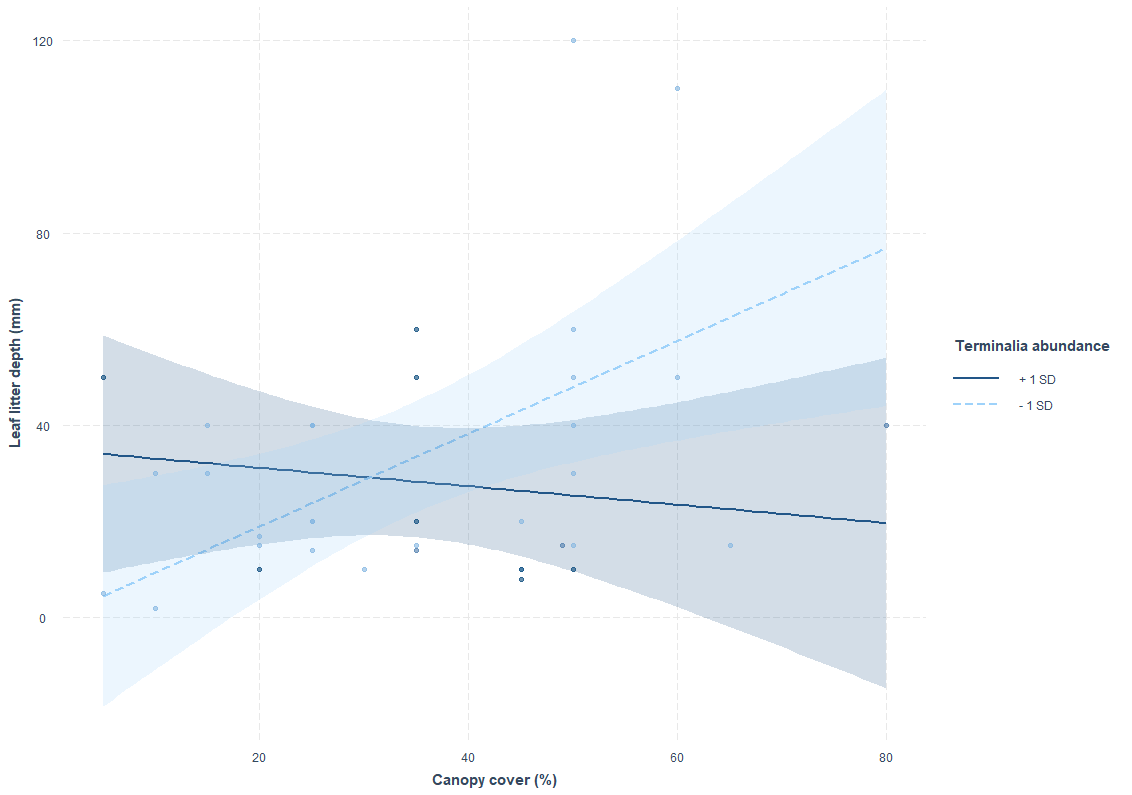


Fig. 3. Interaction plot between the abundance (as measured on a scale of 1-5: 1 = <10% cover, 2 = 10-24.9%, 3 = 25-49.9%, 4 = 50-74.9%, 5 ≥ 75%) of *Terminalia catappa* and canopy cover with respect to leaf litter depth.

**References**

Dargie, T. C. D. 1984. On the integrated interpretation of indirect site ordinations: a case study using semi-arid vegetation in southeastern Spain. Vegetatio **55**:37-55.

Kantvilas, G., and P. R. Minchin. 1990. An analysis of epiphytic lichen communities in Tasmanian cool temperate rainforest. Vegetatio **84**:99-112.

ter Braak, C. J. F. 1986. Canonical Correspondence Analysis: a new eigenvector technique for multivariate direct gradient analysis. Ecology **67**:1167-1179.
